# Application of second-generation sequencing in congenital pulmonary airway malformations

**DOI:** 10.1038/s41598-022-24858-3

**Published:** 2022-11-28

**Authors:** Gang Zhang, Chun Cai, Xiao Li, Lei Lou, Bin Zhou, Huiyi Zeng, Xiangang Yan, Dandan Liu, Gang Yu

**Affiliations:** 1 Department of Pediatric Surgery, The Third Affiliated Hospital of Guangzhou Medical University, No. 63, Duobao Road, Liwan District, Guangzhou City, 510150 Guangdong Province China; 2 Department of Ultrasound Medicine, The Third Affiliated Hospital of Guangzhou Medical University, No. 63, Duobao Road, Liwan District, Guangzhou City, 510150 Guangdong Province China

**Keywords:** Computational biology and bioinformatics, Respiratory tract diseases

## Abstract

To investigate the differential expression of genes in whole transcripts of congenital pulmonary airway malformation (CPAM) using second-generation sequencing (also known as next-generation sequencing, NGS) technology. Children with CPAM were strictly screened after setting the criteria, and grouped by taking CPAM parietal tissue and CPAM lesion tissue respectively, and RNA-Seq libraries were established separately using second-generation sequencing technology, followed by differential expression analysis and GO (gene ontology) functional enrichment analysis, KEGG (Kyoto encyclopedia of genes and genomes, a database) pathway analysis and GSEA (Gene Set Enrichment Analysis) analysis. Five cases were screened from 36 children with CPAM, and high-throughput sequencing was performed to obtain 10 whole transcripts of samples with acceptable sequence quality and balanced gene coverage. One aberrantly expressed sample (3b) was found by analysis of principal components, which was excluded and then subjected to differential expression analysis, and 860 up-regulated genes and 203 down-regulated genes. GO functional enrichment analysis of differentially expressed genes demonstrates the functional class and cellular localization of target genes. The whole transcript of CPAM shows obvious gene up and down-regulation, differentially expressed genes are located in specific cells and belong to different functional categories, and NGS can provide an effective means to study the transcriptional regulation of CPAM from the overall transcriptional level.

## Introduction

Congenital pulmonary airway malformation (CPAM) is the most common type of congenital respiratory malformation, accounting for 30% to 40% of all cases^1^. The prevalence of CPAM ranges from 1/35,000 to 1/7,200, and with the advancement of prenatal diagnosis technology, more and more children with CPAM are being diagnosed prenatally^2,3^. CPAMs are characterized histologically with the presence of multiple cysts in the lung tissue, absence of normal alveoli due to hyperplasia and dilatation of the terminal bronchi. CPAM was first described in 1949^4^ and the now widely used Stoker classification, which classifies CPAM into five types based on histopathology^5^.

The pathogenesis of CPAM is undetermined, but some genes are thought to be possibly linked. L858R point mutation in exon 21 of EGFR (epidermal growth factor receptor) was detected in an 80-year-old case with CPAM^6^. Mutation of thyroglobulin (TG) and its receptor, megalin (LRP2) were found in CPAM patients^7^. Fibroblast growth factor receptor 2 (FGFR2) has been demonstrated by immunohistochemistry in human CPAM cases^8^. Variety of genes have also been implicated in this process of CPAM such as sex-determining region Y- box 2 gene (Sox2), hyroid transcription factor gene (Nkx2), Hox gene (Hoxb-5), fatty acid-binding protein-7 gene (FABP-7) and Ying Yang 1 gene (Yy1)^1,9,10^. In another study, all type 4-CPAM patients possessed the mutation of Dicer 1^11^. In addition, KRAS mutation was demonstrated in the mucinous proliferations and adjacent nonmucinous CPAM tissue^12^. Although many mutations have been identified in CPAM, more unknown genetic alterations exist, and a high-throughput method for detecting genetic alterations in CPAM is particularly urgent.

Second-generation sequencing (SGS), also known as next-generation sequencing (NGS), is a technology which has been widely used to analyze genes for clinical diseases with unprecedented speed and lower price compared to traditional Sanger sequencing^13,14^. NGS technology was developed in 2005 and enables the sequencing of thousands to millions of templates at a time^15^. Therefore, this technology provides the ability to analyze exon mutations and copy number alterations, thereby allowing for the detection of abnormal gene expression in different diseases. NGS analysis identifies COL4A1 and COL4A2 as strong candidates for susceptibility genes in visceral aneurysms^16^. Sivakumar Gowrisankar et al. identified insertion and deletion mutations in 19 genes in Dilated Cardiomyopathy by NGS^17^. NGS has been applied in kidney disease^18^, neurological disease^19^ and several cancers^20^. Although some studies have identified genes associated with congenital pulmonary airway malformations by NGS, the results are not consistent^11,12^, implying that research in this area is still insufficient.

In this study, we aim to find genes and pathways closely related to CPAM by NGS for RNA-seq to provide support for possible subsequent therapeutic options.

## Materials and methods

### Materials

The pediatric surgery department of our hospital admitted a total of 36 children with CPAM in the first quarter of 2020, and the case samples were strictly selected according to the following criteria:

1. The prenatal examination, preoperative CT, intraoperative diagnosis, and postoperative pathological examination of the child were determined to be type 1 CPAM, this type of cyst is usually multicentric and located within a lung lobe with a lining of pseudostratified columnar epithelium, accounts for approximately 70% of all CPAM. This type has an excellent prognosis after resection and rarely undergoes malignant transformation.
2. The children were 0.5–1 years old, with a lesion diameter of 5–10 cm, no edema before birth, no preoperative infection, no chemotherapy or radiation treatment, and no clear combined chromosomal abnormalities.
3. The children were treated with minimally invasive thoracoscopic surgery, the procedure was smooth, no chest tube was left in place after surgery, no infection, bleeding, reoperation, etc. No retention, recurrence, reoccurrence, secondary infection, pneumothorax, etc. were seen at 1 year of follow-up.
4. The study protocol was approved by the Ethics Committee of our hospital (Yi Lun Hui Shen [2020] No. 030). The child guardians signed the informed consent form, clarified the situation of the study and agreed to this study.

### Clinic sample collection

The study protocol was approved by the Ethics Committee of our hospital (Yi Lun Hui Shen [2020] No. 030). All participants received written and oral information prior to giving written consent, and the study was performed in accordance with the Helsinki II declaration. CPAM samples (n = 4), and matched tissue adjacent to CPAM (n = 4) were acquired at the time of surgical resection in the Third Affiliated Hospital of Guangzhou Medical University. All samples were taken from the distal parenchyma away from the hilum. Samples were frozen in liquid nitrogen within 120 s after the sample is isolated and stored at − 80 °C for further analysis. CPAM samples were verified by the Third Affiliated Hospital of Guangzhou Medical University Department of Pathology. More details can be seen in Supplementary Fig. 1.

### RNA isolation and sequencing

Total RNA was isolated from samples using a TRIzol reagent (Invitrogen, Carlsbad, CA, USA). We checked the concentration and purity of RNA by a Nanodrop ND-2000 spectrophotometer (Thermo Fisher Scientific, Wilmington, DE, USA). The standard of RNA is that quantity > 5 µg and concentration ≥ 200 ng/µL. The RNA integrity number (RIN) value (RIN > 7) was tested by an Agilent 2100 Bioanalyzer (Agilent, Palo Alto, CA, USA). Then remove rRNA from total RNA by Ribo-Zero Gold rRNA removal kit (Illumina). After that, RNA was fragmented into size of 200 bp by total RNA SEQ (H/M/R) library prep kit (Illumina, San Diego, CA, USA). The complementary DNA (cDNA) was then synthesized by fragmented RNA. After purification, end repairing and adapter ligation, A-tail adding, connecting product purifying, fragment size sorting and library amplification were performed. After amplification, the RNA SEQ library was obtained purification and recovery with magnetic beads. After inspection of the constructed library, the qualified library was pooled according to the data size and effective cDNA concentration and target data. The library sequenced using the Illumina NovaSeq 6000 platform in paired-end 150-bp mode with a data volume of 10G.

Raw data were filtered to remove low quality reads, and reads containing adapter or Poly-N sequences. The obtained clean reads were mapped to the Ribosomal Database Project with Bowtie (Langmead et al., 2009), and the reads that belonged to ribosomal DNA (rRNA) were removed. Mapping reads to the reference sequence were used for mapping, sequence prediction, expression value calculation and expression difference analysis.

### RNA expression analyses

Principal component analysis (PCA) was done on gene counts to determine the variability in the data set. *P* < 0.05 with a false discovery rate (FDR) q < 0.05 (5%) and a family-wise error rate (FWER) *P* < 0.05 were considered to be statistically significant.

### Gene-set enrichment analysis

For GSEA analysis the online version of the GSEA tool was used. GO functional analysis of DEGs were subdivided into three groups: biological process (BP), molecular function (MF) and cellular component (CC). A *P*-value of less than 0.05 and gene count > 5 was set as the cutoff. A nominal *P* < 0.05 was considered significant.

## Results

### Gene set enrichment analysis

**Figure 1:**
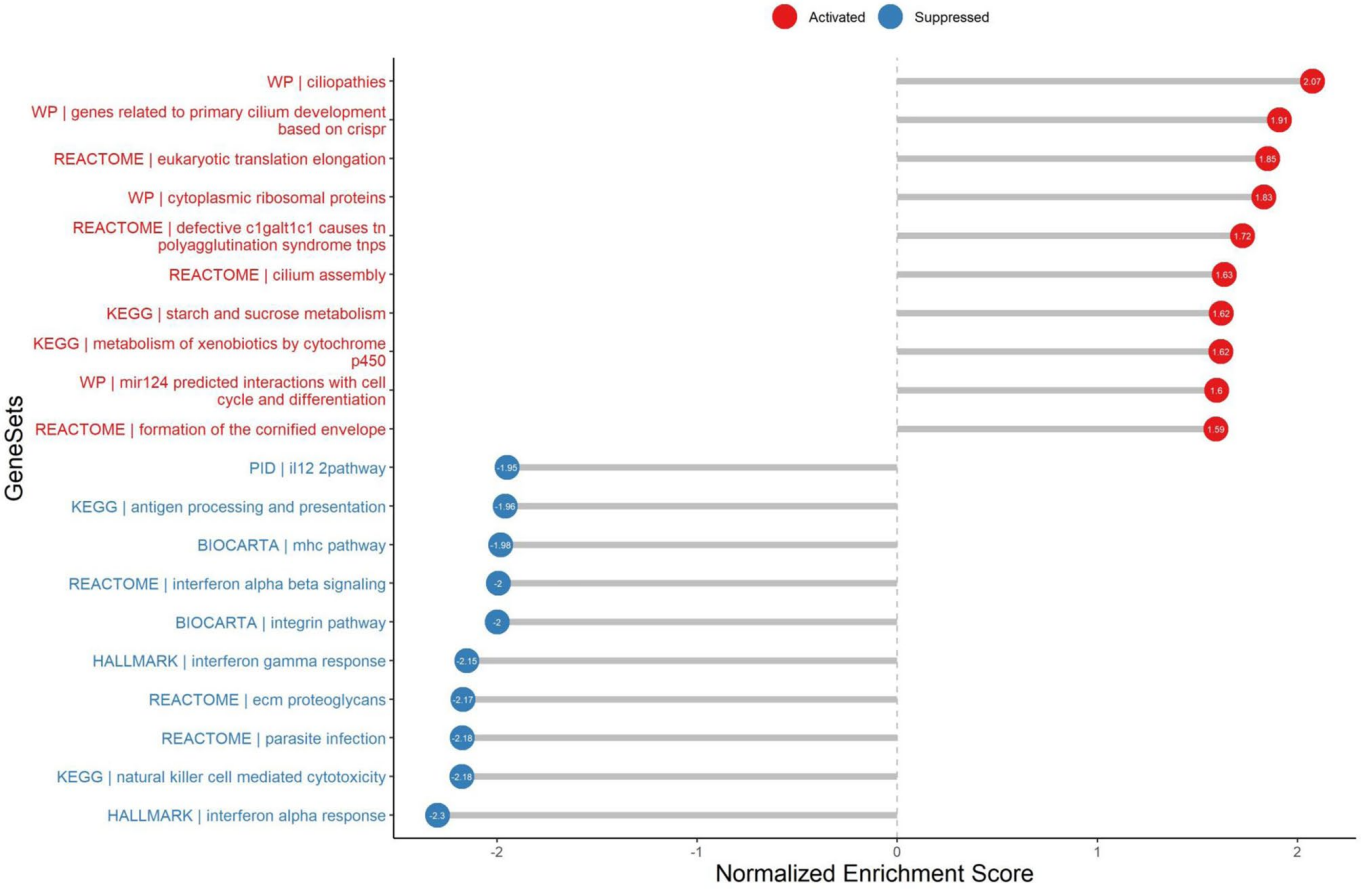
GSEA analys in CPAM parietal tissue vs CPAM lesion tissue.

To identify characteristics specific for the CPAM, we first performed gene set enrichment analysis (*GSEA*: http://www.broadinstitute.org/gsea/index.jsp), the results showed that genes related to primary cilium development, ciliopathies, and cilium assembly were upregulated in CPAM (Fig. 1), which highlights that the appearance of primary cilium in the airway of CPAM. The analysis also showed the significant enrichment in eukaryotic translation elongation, ribosomal proteins, starch and sucrose metabolism pathways, which might associate with energy supplies for cell activation and proliferation. Remarkably, the genes involved in the antigen processing and presentation, mhc pathway, IL-12pathway, interferon gamma response and natural killer cell mediated cytotoxicity were all downregulated (Fig. 1), implying a suppressed innate immune response.

### Identification of DEGs (differently expressed genes)

**Figure 2:**
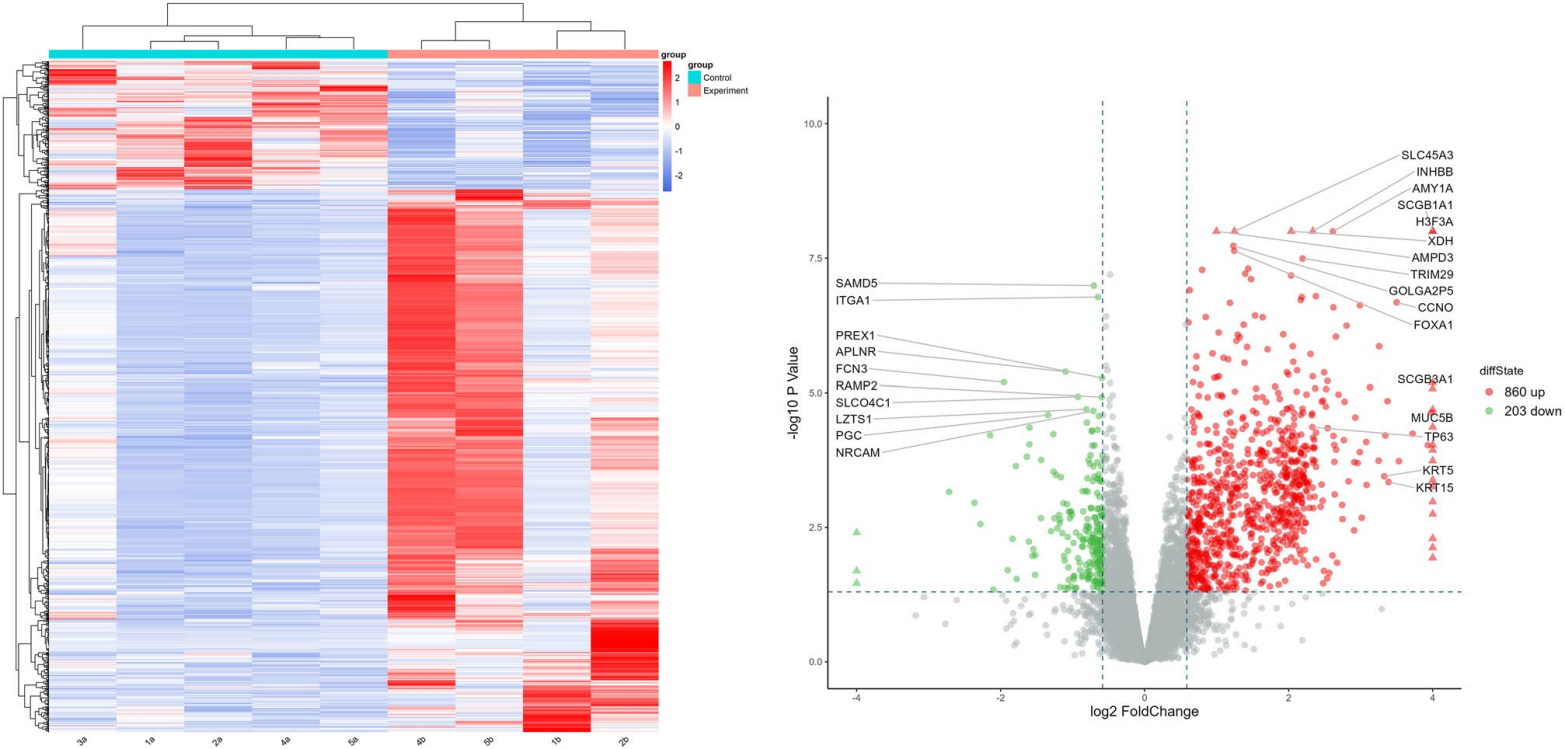
Heatmap and Volcano plot of the differencited expression genes.

Differently expressed genes analysis was performed in succession with the *p* < 0.05, FC > 1.5 and FDR < 1. 742 genes were dysregulated, among them, 130 genes were downregulated and 612 genes were upregulated (Fig. 2). The result showed that H3F3A is the most up-regulated gene, which is not expressed in the control sample. H3F3A regulates chromatin epigenetic modification, and it means that epigenetic modification plays an important part in occurrence and development of CPAM. More interestingly, some epithelial cell marker genes were also increased in CPAM sample such as SCGB1A1, SCGB3A1 (airway secretory cell marker gene)^21,22^; TP63, KRT5, KRT15 (the basal-like cell marker gene)^23^; MUC5B(goblet/cup cell marker gene), and CCNO(the mother centriole) which is important for multiciliated cells assembly^24,25^.

### Functional enrichment analysis of DEGs

**Figure 3:**
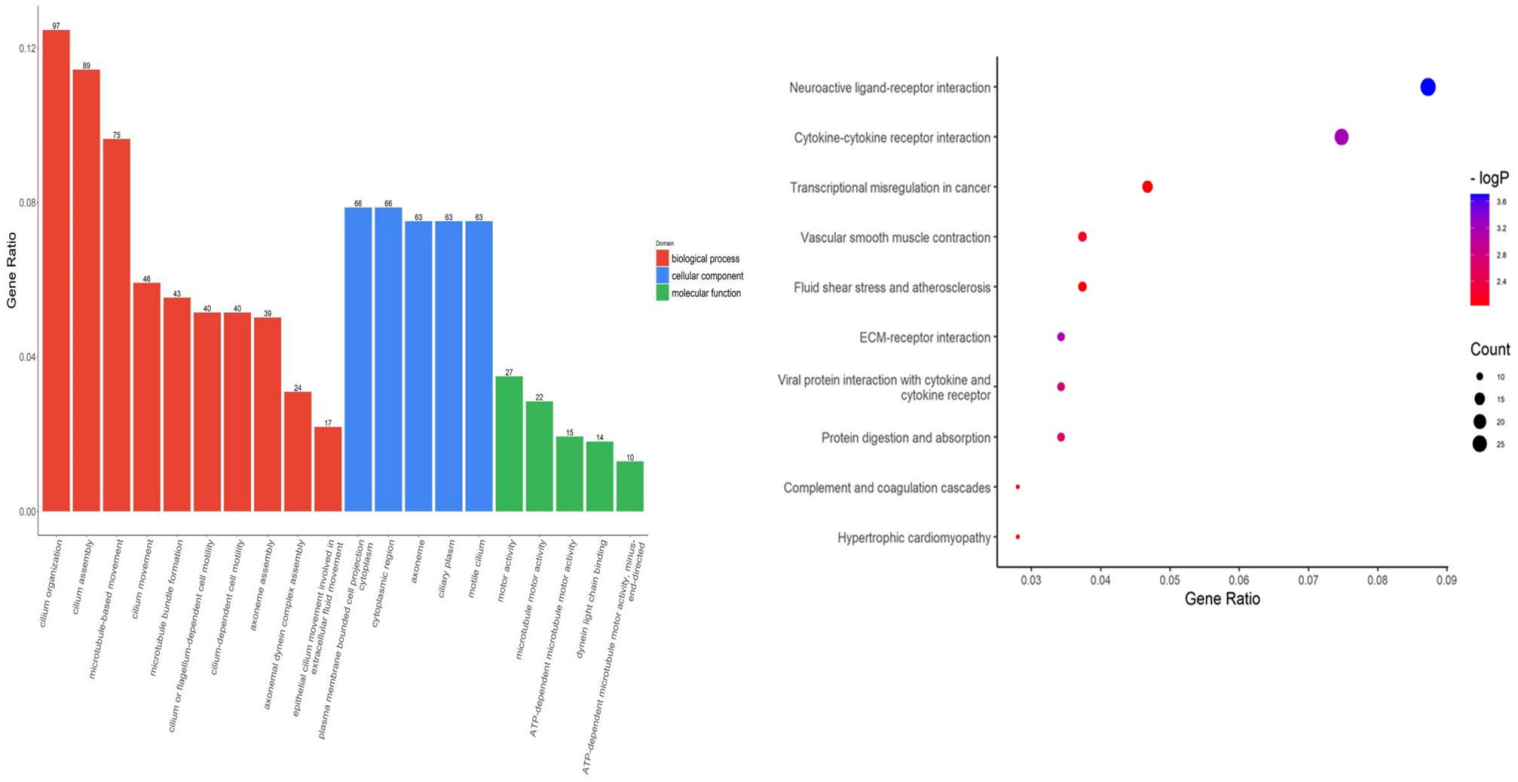
GO and KEGG analyse of DEGS. Left: GO functional analysis for DEGs were grouped into different functional categories: biological process (BF), cellular component (CC) and molecular function (MF). Right: Representative dot plot of top 10 significantly (*P* < 0.05) enriched KEGG pathways for DEGs.

GO analysis results showed that changes in DEGs were enriched for terms related to cilia. Changes in CC (cell component) of DEGs were mainly enriched in the basal body and the axoneme, as well as the transition zone, compartmentalization for signaling and motility functions^26^. Changes in BP (biological processes) of DEGs were mainly enriched in cilium assembly, cilium movement, axoneme assembly. Changes in MF were significantly enriched in ATP-dependent motor activity and dynein chain binding. (Fig. 3). KEGG pathway analysis showed that DEGs were mainly enriched in Neuroactive ligand–receptor interaction and Cytokine–cytokine receptor interaction (Fig. 3). Neuroactive ligand–receptor interaction especially refers to neuropeptide with their receptors, like adrenaline and Calcitonin with the corresponding receptor ADRA1B and CALCRL. Cytokine–cytokine receptor interaction mainly refer to chemokine like CCL11/CCL14/CCL16/CCL19.

### PPI network construction of DEGs

**Figure 4:**
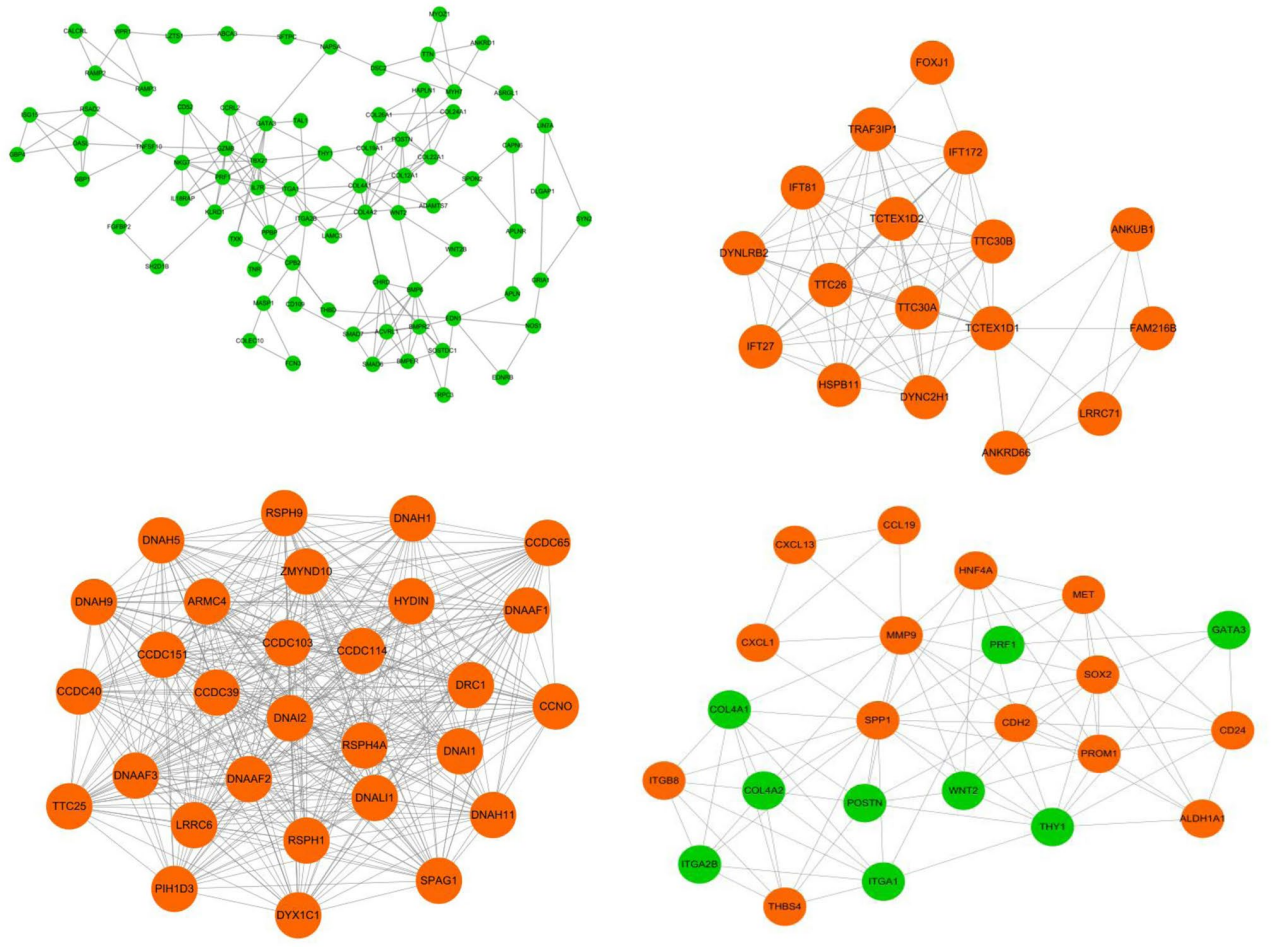
PPI network for DEGs in CPAM. Genes in orange circles indicate upregulation, while genes in green circles indicate downregulation.

To deep excavate the key genes involved in the development of CPAM, we estimate the interaction relationship between DEGs by STRING website. The PPI network was constructed by Cytoscape involved in all the DEGs. The hub cluster was filtered to further analysis (Fig. 4). The down-regulated cluster included BMP6, the receptor BMPR2 and ACVRL1, the signal molecular smad6 and smad7, the auxiliary molecular for BMP like Chrd and BMPER. This cluster shows an integrated suppression of the BMP signaling pathway. WNT2 and WNT2b are also downregulated. WNT signaling maintains proliferation, while the BMP pathway regulates the differentiation. Additionally, the ECM modeling pathway comprise diverse collagen and the metalloproteinase ADAMTS7 which has been reported to fuel BMP2-dependent osteogenic differentiation^27^.

The up-regulated cluster also emphasizes the significance of cilium. The cilium axoneme is maintained by the intraflagellar transport (IFT) machinery which comprises several subcomplexes, including heterotrimeric kinesin and dynein to move IFT complex between the tip and the base^25^. And the hub cluster include DYNC2H1, IFT81, TTC26 associated with IFT complexes and some other genes^26^. Also, DNAH9, CCDC103, DNAI2 and DRC1 are members of multiprotein complex associated with the outer dynein arm complex of motile cilia^26^. Meanwhile, FOXJ1 is a key transcription factor in the development of cilla.

Moreover, some established genes regulating CPAM have also been found in the hub cluster, such as SOX2.SOX2 has been reported as an up-regulated gene leading to CPAM^28^. From the network, the mechanism for SOX2 controlling CPAM might be associated with the stem cell maintenance because the stem cell marker PROM1 concurrently upregulated^29^. Surprisingly,Gata3, a critical regulator for Innate lymphoid cells (ILCs)^30^, was down-regulated in the network, and the same with other immune regulators like PRF1, TBX21, IL7R. This indicates the suppressed development of immune cells residing in CPAM sample. Finally, the up-regulated genes CCL19, CXCL13 and CXCL1 were in accordance with the KEGG enrichment in Cytokine–cytokine receptor interaction pathway.

## Discussion

The etiology of CPAM is not yet well understood, and 2 main hypotheses have been proposed^31^: One of the hypotheses is the obstructive hypothesis, which was proposed by Stocker J^32^. The autopsy performed by Moerman P provides evidence for this hypothesis^33^, they believed that functional or organic obstruction of the bronchi leads to abnormal cell proliferation and apoptosis during lung development. Among the 36 children admitted to our clinic this quarter, 2 had focal obstructions due to combined bronchial cysts. The other is the environmental Hypothesis, which suggests that genetic defects or microenvironmental changes lead to abnormal expression of regulatory factors that result in abnormal lung development. Molecular expression and signaling pathways in endodermal epithelial and mesenchymal cells play a key role during lung development, such as Nkx2-1, the gene encoding thyroid transcription factor (TTF1) in the ventral epithelium of the foregut, Y-box2, a gene in the sex-determining region of the dorsal foregut, and other genes such as Sox2 and Hoxb-5^34^. Instead, BMPs and their antagonists Noggin, FGF10, and Wnt act in mesenchymal cells. When the signaling communication between epithelial cells and mesenchymal cells is disturbed, it leads to impaired alveolar formation in the lung parenchyma and excessive hyperplasia and dilatation of the terminal fine bronchioles, forming single-room, multiroom or cellular misshapen tumor-like CPAM^1^.

With the development of genomics, the occurrence of many diseases has been confirmed to be closely related to genes, and more scholars agree with the view that genes regulate diseases, and how to find the genes that change in diseases has become the basis of studying diseases. Currently, NGS technology can rapidly obtain sequence information of almost all transcripts of a specific organ or tissue of a species in a certain state, and perform differential gene expression analysis, variable shear and fusion gene analysis between disease and normal samples, and then search for disease-causing genes and explore pathogenesis. In the experiment, we successfully obtained 10 sample sequences with balanced gene coverage from 5 pairs of samples in this group using this technique, and successfully constructed RNA-Seq libraries and compared them to the genome. Finally, 860 up-regulated genes and 203 down-regulated genes were obtained. The sequence information of this whole transcript suggested that Nkx2-1 was normally expressed, Hoxb-5 was normally expressed and Sox2 was highly expressed, and the results were the same as those of other scholars^35^. BMPs belong to the TGF-β family, and several BMPs and their receptor BMPR play regulatory roles in lung development^1,35^. In our group, we found that BMP8A and BMPR1B expression was upregulated, BMP6, BMPR2 and BMPER (Homo sapiens BMP binding endothelial regulator, mRNA.) expression was downregulated, and BMPR1A expression was unchanged.

To clarify the functions of differentially expressed genes, pathway enrichment analysis can be performed using the R software clusterProfiler with functional gene collections^36^. In this paper, we choose the Gene Ontology (GO) collection, which can be divided into three parts: molecular function, biological process and cellular component. In functional enrichment analysis, high expression of DNAAF4, FOXJ1, LRP2, MNS1, and WT1 was found, suggesting a close relationship between CPAM and abnormal lung development^37^, while SOX2, a transcriptional regulator, was involved in BCAS1, CDH2, COMMD3-BMI1, CYP2J2, FBXO15, KIF19, KRT17, PROM1, TNR and other regulators^35^. Rawlins EL^38^ found that FOXJ1 is progressively expressed in ciliated cells during lung development and that when FOXJ1 is fully expressed in ciliated cell precursors, the cells stop proliferating or have a significantly longer division cycle. Differential expression analysis in this group of samples revealed high FOXJ1 expression (*P* = 0.00024), and GO functional enrichment analysis suggested that FOXJ1 is involved in various aspects of respiratory differentiation, respiratory epithelial cells, ciliary cell differentiation, cilia assembly and motility^39^.

Of course, there are limitations in our study. After all, NGS has two major drawbacks^40^: shorter reads and lower accuracy compared to Sanger sequencing, so it would be better if genes abnormally expressed in NGS sequencing could be validated by transcription or translation test. Moreover, if we get a larger sample size, the results may be more reliable.

In summary, whole transcriptome sequencing using NGS technology can obtain stable and reliable RNA-Seq libraries of CPAM patients, and differential expression analysis suggests significant gene differences between lesioned and paralesional tissues, with significant gene up- and down-regulation in lesioned tissues. GO-functional analysis of the differentially expressed gene collection could initially locate the functional class and cellular localization of each gene. Thus, NGS provides an effective means to study the transcriptional regulatory network and to screen molecular biological markers of CPAM.

## Supplementary Information

Supplementary Information.

## Data Availability

The datasets generated and/or analysed during the current study are available in GEO website with the accession number: GSE190620.
