# Supplementary material for: Application of second-generation sequencing in congenital pulmonary airway malformations
Source: Sci Rep. 2022 Nov 28;12:20459. doi: 10.1038/s41598-022-24858-3 (PMC9705386; doi:10.1038/s41598-022-24858-3)
Supplement: Supplementary file 1 — Supplementary Information. [file 41598_2022_24858_MOESM1_ESM.docx]

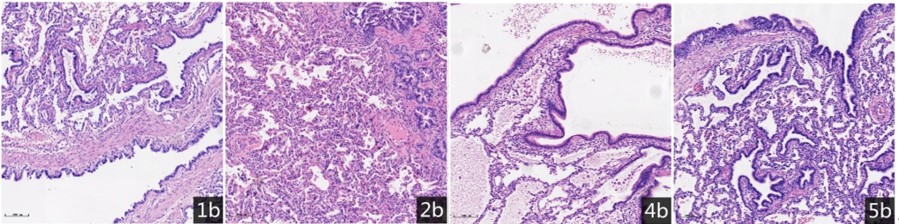


Supplementary Figure 1 CPAM samples(1b-5b) are type 1.  More than one cyst with thick walls involve the single lobe. The cyst wall is mainly composed of pseudostratified ciliated columnar epithelium microscopically(10×).


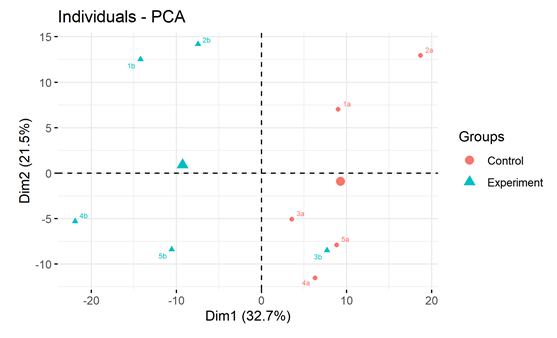


Supplementary Figure 2 PCA of samples gene expression. The gene expression of 3b is abnormal.
